# Exploring the displacement characteristics of Garden III femoral neck fractures and the reliability, validity, and value of the anteroposterior Garden Index in assessing displacement severity

**DOI:** 10.1186/s13018-023-04269-4

**Published:** 2023-10-24

**Authors:** Bo Cong, Ziyin Han, Haiguang Zhang

**Affiliations:** Yantai Key Laboratory for Repair and Reconstruction of Bone and Joint, Yantaishan Hospital Affiliated to Binzhou Medical University, 10087 Keji Avenue, Laishan District, Yantai, 264003 China

**Keywords:** Femoral neck fractures, Garden III, Anteroposterior Garden Index, Displacement, Computed tomography (CT), Reliability, Validity, Fracture endpoint, Contact surface area

## Abstract

**Background:**

Femoral neck fractures represent a significant public health concern, particularly in the elderly population. A thorough understanding and assessment of these fractures are deemed essential for optimal treatment and management. Displacement characteristics of Garden III femoral neck fractures were explored in this study, and the reliability, validity, and clinical utility of the anteroposterior Garden Index in evaluating displacement severity were investigated.

**Methods:**

Patients diagnosed with Garden III femoral neck fractures were included in this study. The anteroposterior Garden Index was computed from X-ray images by three experienced orthopedic doctors. Additionally, the contact area of the fracture endpoint and displacement of the femoral neck were evaluated using 128-slice 3D CT scans. Inter-observer and retest reliability of the Garden Index measurements were assessed, along with its correlation with CT measurements.

**Results:**

In this study, a total of 110 patients with Garden III femoral neck fractures were analyzed, showcasing an almost equal gender distribution and an age range spanning from 20 to 88 years. An average Garden Index of 135° (± 16°) was observed. The intra-observer repeatability of the Garden Index was found to exceed 90%. A significant positive correlation was identified between the Garden Index and the contact surface area of the fracture endpoint (*r* = 0.82, *P* < 0.001), while a significant negative correlation was noted with the upward displacement of the femoral neck (*r* = − 0.79, *P* < 0.001).

**Conclusions:**

The anteroposterior Garden Index has been demonstrated to have promising potential as a reliable and valid tool for assessing the displacement severity of Garden III femoral neck fractures. Nonetheless, further research is needed to elucidate its relationship with other fracture characteristics and to enhance its criterion and construct validity.

## Introduction

Femoral neck fractures, primarily seen in the elderly, are estimated to constitute 48% to 54% of hip fractures. These injuries pose a significant therapeutic challenge due to complications like non-union and avascular necrosis of the femoral head [1, 2]. Challenges arise from various factors, including the degree and type of fracture displacement, quality of reduction, nature of injury force, patient age, bone density, and comorbidities [3, 4]. The Garden classification, based on the severity of fracture displacement, is the most widely used clinical classification for these fractures. Femoral neck fractures are categorized into four types using this classification: Type I (incomplete fracture), Type II (complete fracture with no displacement), Type III (complete fracture with partial displacement), and Type IV (complete fracture with total displacement). Type I, II, and IV fractures are characterized by a single degree of displacement (either none or total), while Type III fractures display a range of displacements, from none to total [5].

Significant variability in the degree of displacement in Garden III fractures has been observed, with corresponding variations noted in the extent of damage to the femoral head blood supply and outcomes after internal fixation. This variability suggests a potential correlation between the prognosis of Type III fractures and their degree of displacement. At present, no reliable, accurate, convenient, and practical method has been established to quantitatively assess the displacement degree of Type III fractures. Optimal treatment options for Garden Type III femoral neck fractures in patients aged 55 to 75 years, be it internal fixation or total hip arthroplasty, continue to be debated due to a lack of objective and scientific decision-making criteria [6, 7]. The Anteroposterior (AP) Garden Index serves as an indicator to evaluate the quality of fracture reduction and prognosis, with its values indicating the position of the fracture end following internal fixation. Yet, it remains to be determined whether the AP Garden Index can accurately differentiate the fracture end position status of Type III femoral neck fractures with varying degree of displacement. 

In light of the variability observed in Garden III femoral neck fractures and the challenges encountered in clinical decision-making, this study seeks to provide a more detailed understanding of the displacement characteristics of these fractures. The reliability, validity, and value of the AP Garden Index as a potential tool in determining the severity of displacement are assessed. The intention of this research is not to propose a definitive solution to the issue of fracture variability, but rather to contribute to a more informed basis for treatment decision-making. By providing a clearer perspective on the nature of Garden III fractures and the potential applicability of the AP Garden Index, it is anticipated that orthopedic surgeons can tailor their treatment strategies more effectively, thereby enhancing patient outcomes. It remains crucial to note that, while this study seeks to advance understanding, it represents a step in the ongoing journey of optimizing patient management for Garden III fractures.

## Methods

### Patient selection

Patients with Garden III femoral neck fractures were included, without gender restriction, aged over 18 years, and had undergone an anterior–posterior pelvis X-ray upon admission. Exclusion criteria were as follows: (1) diagnosis contested by three experienced orthopedic doctors, (2) presence of concomitant acetabulum, pelvis, or femur fractures, (3) detection of femoral head necrosis, (4) identification of hip joint malformation or developmental anomalies, and (5) occurrence of pathological fractures.

Diagnostic criteria for Garden III femoral neck fractures are defined as a complete fracture of the femoral neck with partial displacement, internal rotation of the fractured femoral head, mild upward and external rotation of the femoral neck, and discontinuity of trabecular lines between the intra-articular pressure bone of the femoral head and the acetabulum. Informed consent was obtained from all participants, and the study protocol received approval from the Ethics Committee of Yantaishan Hospital.

### Measurement methods and observation indices

**Fig. 1:**
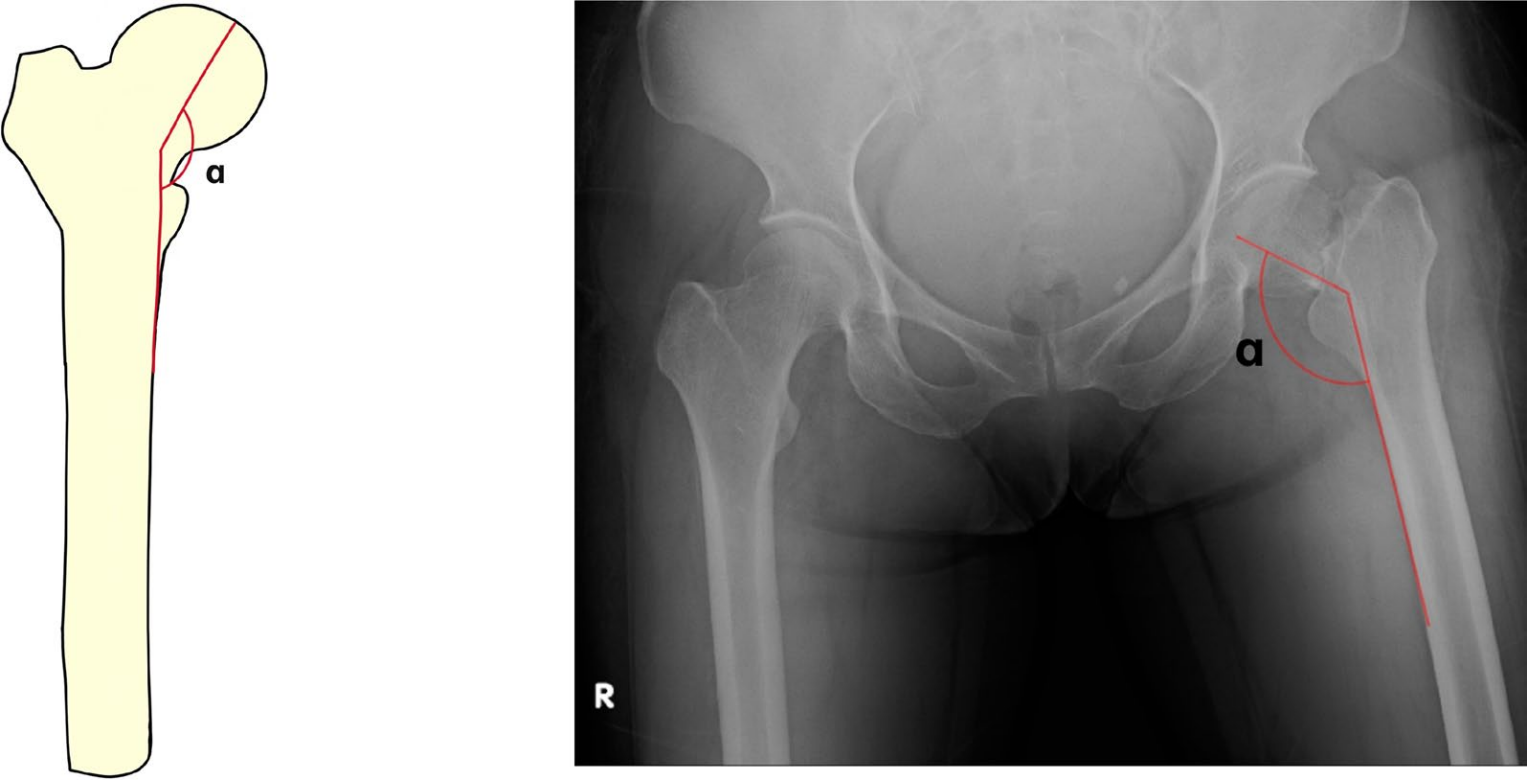
Measurement of the Garden Index: (Left Section) Schematic Diagram: This panel illustrates the methodology employed for measuring the Garden Index. The angle under scrutiny is denoted by the Greek letter α. The angle is measured between the central axis of the trabeculae on the medial side of the femoral neck and the cortical line on the medial side of the femoral shaft. (Right Section) Preoperative Anteroposterior Radiograph: This panel shows an actual preoperative anteroposterior X-ray image of a hip with a femoral neck fracture. The angle in question is similarly labeled as *α*

Garden Index in the anteroposterior view: the Garden Index is calculated as the angle between the central axis of the trabeculae on the medial side of the femoral neck and the cortical line on the medial side of the femoral shaft in an anteroposterior hip X-ray (Fig. 1). The Garden Index of the affected side was measured twice consecutively by three experienced orthopedic doctors (designated as A, B, and C). Three measurements for each patient were taken by each doctor, and the average of their individual measurements was determined. Subsequently, the averaged measurements of all three doctors were calculated. This procedure was then repeated for a second round of measurements.

Fracture Endpoint Contact Area and Femoral Neck Displacement: Using 128-slice 3D CT scans, the contact length of the fracture endpoints on the coronal surface was determined as the number of slices in which the fracture endpoints contacted each other multiplied by the slice thickness. A similar method was employed in the coronal sequence to determine the contact length of the fracture endpoints in the horizontal plane. Given the cross section of the femoral neck is approximately elliptical, the contact surface of the fracture endpoint was also deemed approximately elliptical. As a result, the CT contact area of the fracture endpoint, S, was approximated as 1/2 times the coronal contact length of the fracture endpoint multiplied by 1/2 times the horizontal contact length of the fracture endpoint multiplied by π. The displacement of the femoral neck was determined in the horizontal sequence by the number of slices in which the distal fracture endpoint was observed to be displaced upwards, multiplied by the slice thickness.

### Statistical analysis

Statistical analyses were performed using SPSS 21.0. The Kolmogorov–Smirnov test was used for normality testing of the data. Quantitative data that followed a normal distribution are represented as *x̄* ± *s*. The Spearman correlation coefficient was used for consistency testing of the retest reliability of the Garden Index, with a coefficient over 0.9 considered reliable. The Kappa coefficient[8] was used for inter-observer consistency testing, with a coefficient over 0.8 considered reliable. All tests were two-sided, and a *p* value < 0.05 was considered statistically significant.

## Results

### Participant demographics and clinical characteristics

In this study, a total of 110 patients diagnosed with Garden III femoral neck fractures were analyzed. The gender distribution was nearly equal, with 58 males (52.7%) and 52 females (47.3%). Patient ages ranged from 20 to 88 years, with an average age of 65.6 years and a standard deviation of 18.8 years. Regarding injury causes, falls were the predominant cause, accounting for 68 cases (61.8%), followed by traffic injuries with 16 cases (14.5%). Falls from heights, blows, and strikes by heavy objects accounted for 9 (8.2%), 11 (10.0%), and 6 (5.5%) cases, respectively. The presence of comorbid conditions in the cohort was also documented: hypertension was identified in 39 patients (35.5%), coronary heart disease in 26 (23.6%), chronic respiratory diseases in 16 (14.5%), osteoporosis in 18 (16.4%), and diabetes in 12 (10.9%) (Table 1).

**Table 1:** Demographic and clinical characteristics of patients with Garden III femoral neck fractures

Participant Characteristics	Number of cases	Percentage (%)
Gender		
Males	58	52.7
Females	52	47.3
Age range (years)		
Minimum	20	–
Maximum	88	–
Mean (± SD)	65.6 (± 18.8)	–
Cause of injury		
Falls	68	61.8
Traffic injuries	16	14.5
Falls from heights	9	8.2
Blows	11	10.0
Heavy object strikes	6	5.5
Comorbidities		
Hypertension	39	35.5
Coronary heart disease	26	23.6
Chronic respiratory diseases	16	14.5
Osteoporosis	18	16.4
Diabetes	12	10.9
Patients with 2 or more comorbidities	28	25.5

### Characteristics and consistency of Garden Index measurement in femoral neck fractures

In this study, the distribution characteristics of the Garden Index in the orthostatic position, which refers to the upright or standing position of the patient, and its inter-rater reliability in patients with Garden type III femoral neck fractures were examined. It was found that the orthostatic Garden Index among 110 patients with Garden type III femoral neck fractures exhibited a normal distribution (*Z* = 0.86, *P* = 0.456), with a mean of 135° (± 16°). The minimum recorded value was 89°, the 25th percentile stood at 126°, the median was 138°, the 75th percentile was 148°, and the maximum value reached was 159°. To evaluate the reliability of these measurements, the reproducibility of the Garden Index was analyzed. When measured twice by the same observer, over 90% repeatability of the results was observed (Table 2). Moreover, the consistency of the Garden Index between different observers was assessed. The Kappa values for consistency were calculated as follows: between observers A and B, it was 0.86; between observers B and C, it was 0.92; and between observers A and C, it was 0.88 (all *P* < 0.001). These data suggest that over 80% repeatability was observed in the measurements across different observers. In conclusion, the results indicate a well-defined pattern of the Garden Index distribution in Garden type III femoral neck fractures. Furthermore, both intra- and inter-observer measurements of the Garden Index demonstrated high levels of repeatability, underscoring its reliability as a measurement tool.

**Table 2:** Reliability of the Garden Index measurement among three observers for 110 patients with Garden III type femoral neck fractures (°)

	Observer A	Observer B	Observer C
Min value (1st measure)	91	91	91
Min value (2nd measure)	91	91	91
Max value (1st measure)	158	158	158
Max value (2nd measure)	158	156	158
Mean (1st measure)	137	137	137
Mean (2nd measure)	137	137	137
Std deviation (1st measure)	14	14	14
Std deviation (2nd measure)	12	13	14
Spearman correlation coefficient (*P* < 0.001)	0.94	0.98	0.96

### Validity of Garden Index position in Garden III femoral neck fractures

In this analysis, the content validity of the Garden Index Position in patients with Garden III femoral neck fractures was evaluated. Using data from 56 participants, the Garden Index Position was compared to both the contact surface area of the fracture endpoint on CT scans and the upward distance of the femoral neck on CT scans (refer to Table 2 for details). A positive correlation was found between the Garden Index Position and the contact surface area of the fracture endpoint, with a correlation coefficient of 0.82 (*P* < 0.001). Conversely, a negative correlation was identified between the Garden Index Position and the upward distance of the femoral neck on CT scans, with a correlation coefficient of − 0.79 (*P* < 0.001). These findings suggest that the Garden Index Position might serve as a reliable measure in the assessment of Garden III femoral neck fractures, correlating significantly with key parameters visible in CT scans. These results are detailed in Table 3.

**Table 3:** Measurement results and correlation coefficients for Garden Index position, fracture endpoint contact surface area, and upward distance of femoral neck in patients with Garden III femoral neck fractures

Metric	Garden Index position (°)	Fracture endpoint contact surface area (mm^2^)	Upward distance of Femoral Neck (mm)
Minimum	103	86	7
Maximum	160	768	29
Mean	134	294	18
Standard deviation	19	194	6
Correlation coefficient	–	0.82	− 0.79
*P* value		< 0.001	< 0.001

## Discussion

Femoral neck fractures and their subsequent prognosis are substantially influenced by both their classification and the extent of fracture displacement. This research delves into Garden III type femoral neck fractures, examining the varied degrees of displacement they present and exploring their implications for treatment decisions and prognostic evaluations [9]. Computed tomography (CT) and three-dimensional reconstruction techniques have emerged as dependable tools for a comprehensive assessment of fracture displacement. These innovations grant a clearer understanding of fracture alignment and position and have notably improved the diagnosis and management of femoral neck fractures in three pivotal ways [10].First, they allow for accurate identification of complete fractures and their displacement, thereby improving the diagnostic precision of the Garden classification. Next, they support the creation of new assessment indices and a CT-based classification system for femoral neck fractures [11, 12]. Finally, they provide enhanced visualization of spatial and rotational displacements, which are often undetectable with conventional X-ray imaging.

However, the utility of these techniques for a quantitative assessment of the displacement degree in Type III fractures remains largely unexplored. This gap is accentuated by the fact that the Garden classification, rooted in X-ray examination, doesn't align seamlessly with these advanced imaging methods. Moreover, the evaluative indices employed to characterize fracture displacement often require intricate calculations, many of which are heavily dependent on imaging software or digital models [13]. At present, definitive evidence supporting the use of these technologies to determine the extent of femoral neck fracture displacement for guiding clinical treatment strategies and prognostic estimates is lacking [14, 15]. Consequently, the practicality and ease of these methods in a clinical setting are still debatable.

Our research examined a total of 110 patients diagnosed with Garden III femoral neck fractures. We observed an almost equal gender distribution, with 58 males (52.7%) and 52 females (47.3%), hinting at a balanced susceptibility across genders to this specific fracture type. The patient age spanned a wide range, from 20 to 88 years, with a mean age of 65.6 years. This spread across different age groups, notably the mean, aligns with the understanding of the elevated susceptibility of the elderly to Garden III femoral neck fractures, likely due to age-related bone fragility and an enhanced risk of falls. Pertaining to injury causation, falls were predominant, constituting 68 cases (61.8%), followed by traffic-related injuries with 16 cases (14.5%). Less frequent causes included falls from heights, blows, and heavy object strikes accounting for 9 (8.2%), 11 (10.0%), and 6 (5.5%) cases, respectively. Furthermore, hypertension was the most prevalent comorbidity in our patient sample, seen in 39 out of the 110 patients (35.5%), trailed by coronary heart disease, chronic respiratory diseases, osteoporosis, and diabetes.

Shifting our focus to the Garden Index, our study revealed intriguing insights. Specifically, the degree of displacement in Garden III femoral neck fractures demonstrated a diverse, yet standard distribution. There was a pronounced tilt toward an orthostatic Garden Index of 137°. This data implies that the orthostatic Garden Index is a potent measure, potentially reflecting the displacement degree in these fractures with precision. Leveraging CT and three-dimensional reconstruction technology, we ascertained the reliability of our displacement assessment approach through stringent evaluation [16]. Our research design included exhaustive intra-rater and inter-rater assessments, comprising repetitive measurements by the same evaluator and juxtaposing measurements recorded by different evaluators. This comprehensive validation minimizes potential biases or inaccuracies. Notably, our results unveiled remarkable consistency and reproducibility: intra-rater and inter-rater repeatability surpassed 90% and 80%, respectively. Such impressive repeatability accentuates the orthostatic Garden Index's reliability as a valuable tool for quantifying displacement in Garden III femoral neck fractures. Consequently, varied clinicians employing this index could likely arrive at analogous conclusions regarding displacement degree, fostering uniform assessments and coherent treatment regimens.

The reliability of the orthostatic Garden Index, a crucial feature of any measurement instrument, was underscored by its substantial correlations with other key parameters. Specifically, significant associations were observed between the index and both the CT contact surface area of the fracture endpoint and the upward displacement distance of the femoral neck as documented on CT [17]. These robust relationships validate the orthostatic Garden Index's capacity to accurately represent the genuine displacement in Garden III fractures. Such precise representation of fracture displacement is indispensable for accurate diagnosis, formulation of treatment strategies, and possible prediction of clinical outcomes. Collectively, these insights suggest that the orthostatic Garden Index has potential as a beneficial tool for medical professionals. By offering an accurate depiction of fracture displacement, it can shape therapeutic choices and assist in forecasting the prognosis for patients with Garden III femoral neck fractures.

Despite the significant insights gained from this research, certain limitations are present. The study's emphasis on the displacement of Garden III femoral neck fractures does not fully encompass associated posterior tilt and rotational displacement. Comprehensive exploration of these elements in relation to the orthostatic Garden Index is warranted in subsequent studies. Moreover, the assessment of the orthostatic Garden Index's validity was restricted to content validity. Both its criterion and construct validities necessitate additional clinical evaluation. Future investigations addressing these facets would ensure a more holistic understanding of the orthostatic Garden Index's relevance.

## Conclusions

In conclusion, our study illuminates the potential of the orthostatic Garden Index as a valuable tool in assessing displacement severity of Garden III femoral neck fractures. The high reliability and significant correlations with other relevant measures show promise for its use in clinical settings. However, further research is needed to fully understand its relationship with posterior tilt and rotational displacement and to further validate its criterion and construct validity.

## Data Availability

The datasets used and/or analyzed during the present study are available from the corresponding author on reasonable request.
